# Supplementary material for: Senataxin resolves RNA:DNA hybrids forming at DNA double-strand breaks to prevent translocations
Source: Nat Commun. 2018 Feb 7;9:533. doi: 10.1038/s41467-018-02894-w (PMC5803260; doi:10.1038/s41467-018-02894-w)
Supplement: Supplementary file 1 — Supplementary Information [file 41467_2018_2894_MOESM1_ESM.pdf]

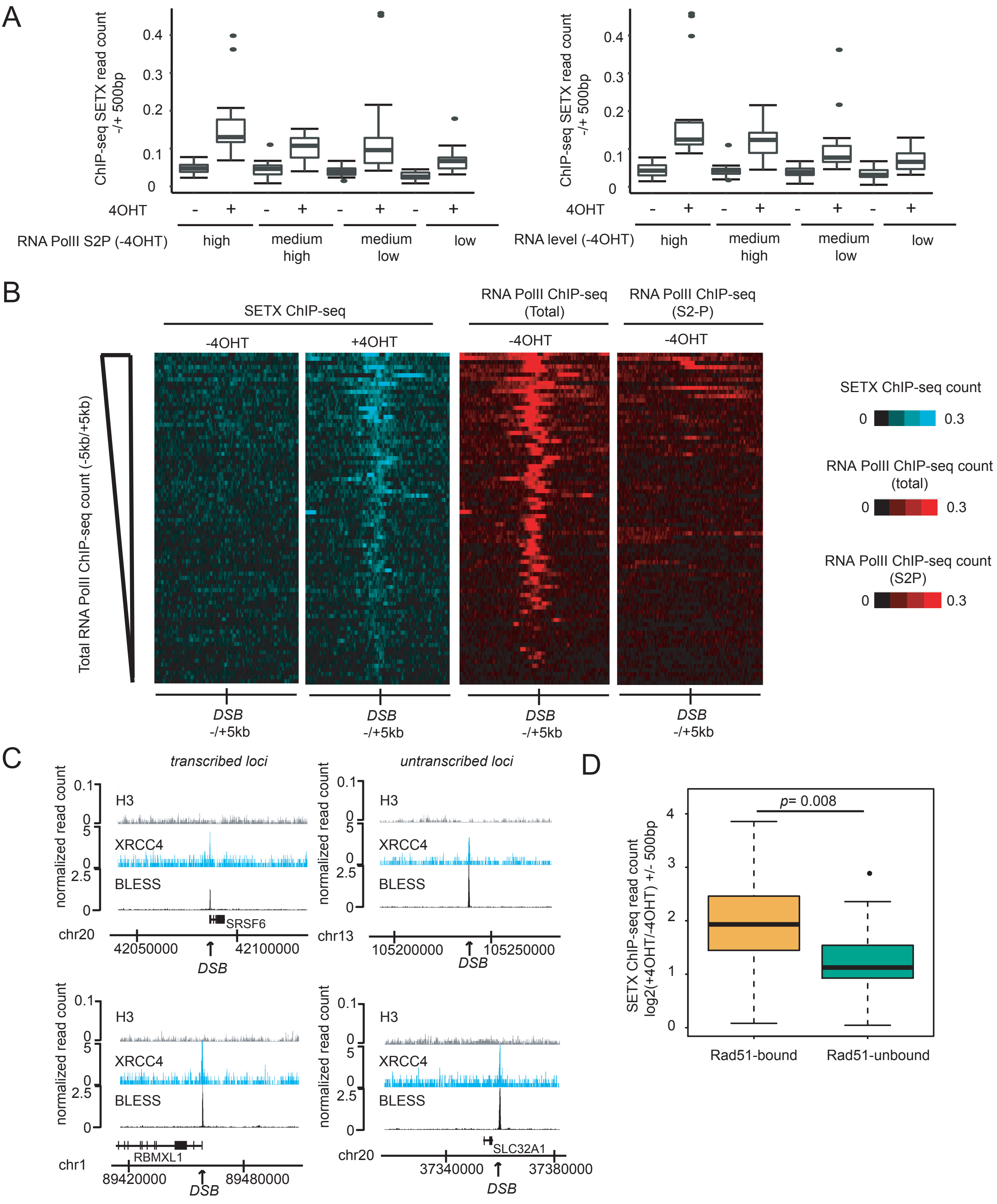

Supplementary Figure 1, related to Figure 1: Senataxin binds to DSBs induced in transcriptionally active loci

A. Box plots representing senataxin ChIP-seq count before (-4OHT) and after (+4OHT) DSB induction at AsiSI "cut" sites sorted according to RNA Polymerase II-S2P occupancy (left panel) or RNA level (right panel) on a 10kb window surrounding AsiSI sites (20 DSBs in each categories). Center line: median; Box limits: 1st and 3rd quartiles; Whiskers: Maximum and minimum without outliers. Points: outliers. **B.** Heatmaps representing SETX ChIP-seq count over a 10kb window centered on the DSB before (-4OHT) and after (+4OHT) DSB induction, as well as RNA Pol II ChIP-seq count prior to DSB induction. DSBs are sorted according to decreasing RNA Polymerase II occupancy. **C.** Genome browser screenshots representing BLESS signal (Clouaire et al, in revision) H3 (negative control, Clouaire et al, in revision) and XRCC4 (positive control, Aymard et al, 2014) ChIP-Seq reads count after damage induction (+4OHT) at the four individual AsiSI sites presented Fig. 1E. Note that despite no senataxin recruitment, the two untranscribed loci display strong recruitment of XRCC4. **D**. Box plot showing the senataxin read count on -/+ 500bp windows at DSBs that exhibit high level of Rad51 (Rad51 bound, 20 DSBs), or low level of Rad51 (Rad51-unbound, 20 DSBs). Data are expressed in log2 (+4OHT/-4OHT). Center line: median; Box limits: 1st and 3rd quartiles; Whiskers: Maximum and minimum without outliers. Points: outliers. *P*-values are indicated (Wilcoxon Mann-Whitney test).

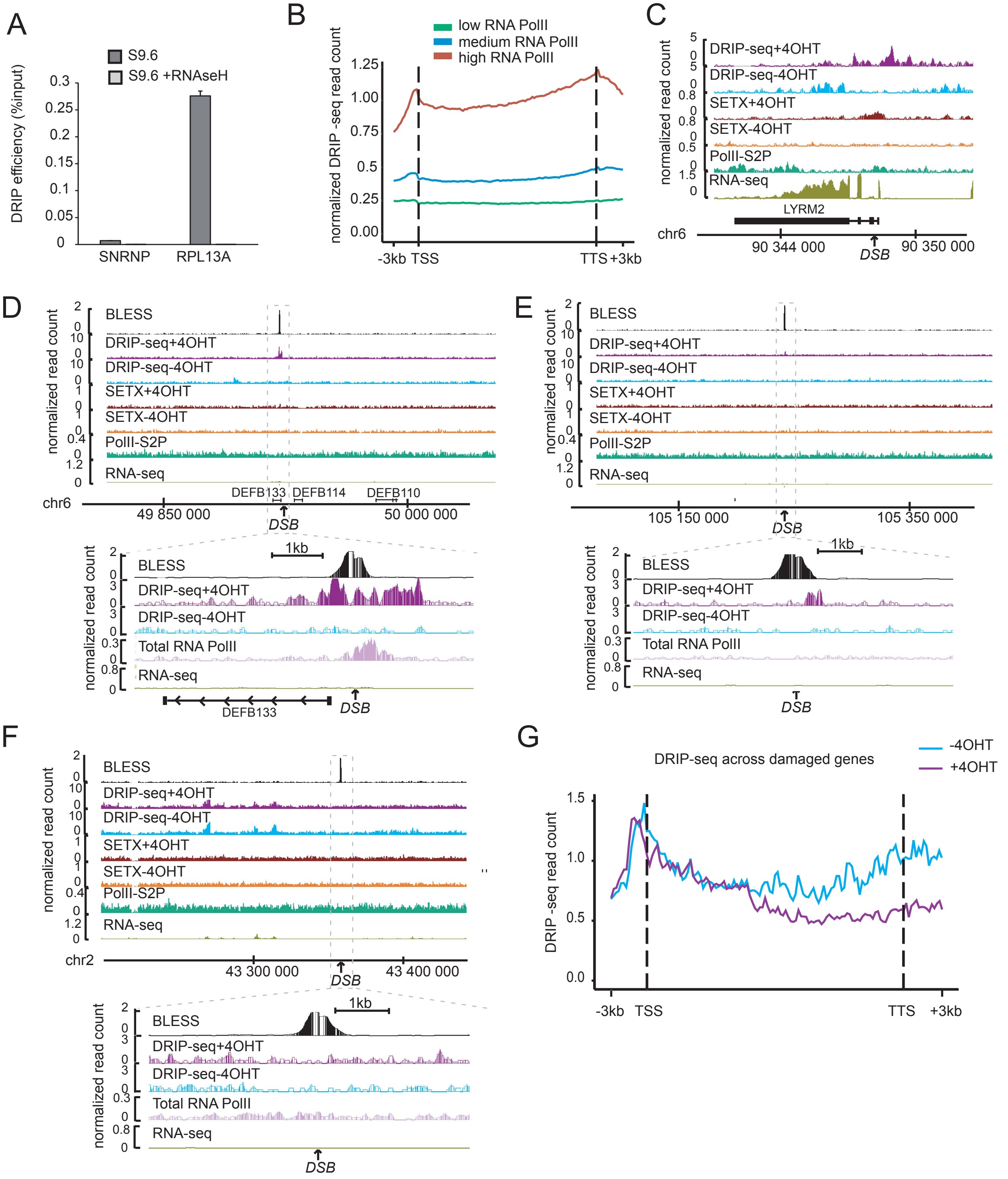

Supplementary Figure 2, related to Figure 2: RNA:DNA hybrids distribution analysed by DRIP-seq in DIvA cells prior and after DSB induction

A. DRIP-qPCR performed in DIvA cells prior DSB induction in presence or absence of RNAseH treatment as indicated, on two genomic loci known to either be devoid (SNRNP) or enriched (RPL13A) in R-Loops. Mean and s.e.m of technical replicates of a representative experiment is shown. **B.** Average DRIP-seq profiles across all genes on the genome (hg19) divided in three categories based on their RNA PolII enrichment across the gene body (high, medium, low, as indicated). **C.** Genome browser screenshot representing DRIP-seq and senataxin ChIP-Seq reads count before (-4OHT) and after damage induction (+4OHT) at a DSB induced in the first intron of a transcribed gene **D.** Genome browser screenshot representing DRIP-seq and senataxin ChIP-Seq reads count before (-4OHT) and after damage induction (+4OHT) at an untranscribed AsiSI site (see RNA PolIIS2P and RNA-seq signals). The BLESS signal (indicative of cleavage efficiency) is also shown. A close-up with Total RNA PolII enrichment is shown on the bottom panel. Note that at this locus, although not transcribed to a detectable level, total RNA PolII is present prior break induction. A low amount of RNA:DNA hybrids forms following DSB induction. **E**. Same as in C, except that total RNA PolII is not detected prior break induction. **F.** Same as in C, except that at this untranscribed locus, no RNA:DNA hybrids forms following breakage. **G**. Average DRIP-seq profiles across the genes either directly damaged (AsiSI within their gene body) or lying at the immediate vicinity of an AsiSI site (<1kb), before and after 4OHT treatment as indicated.

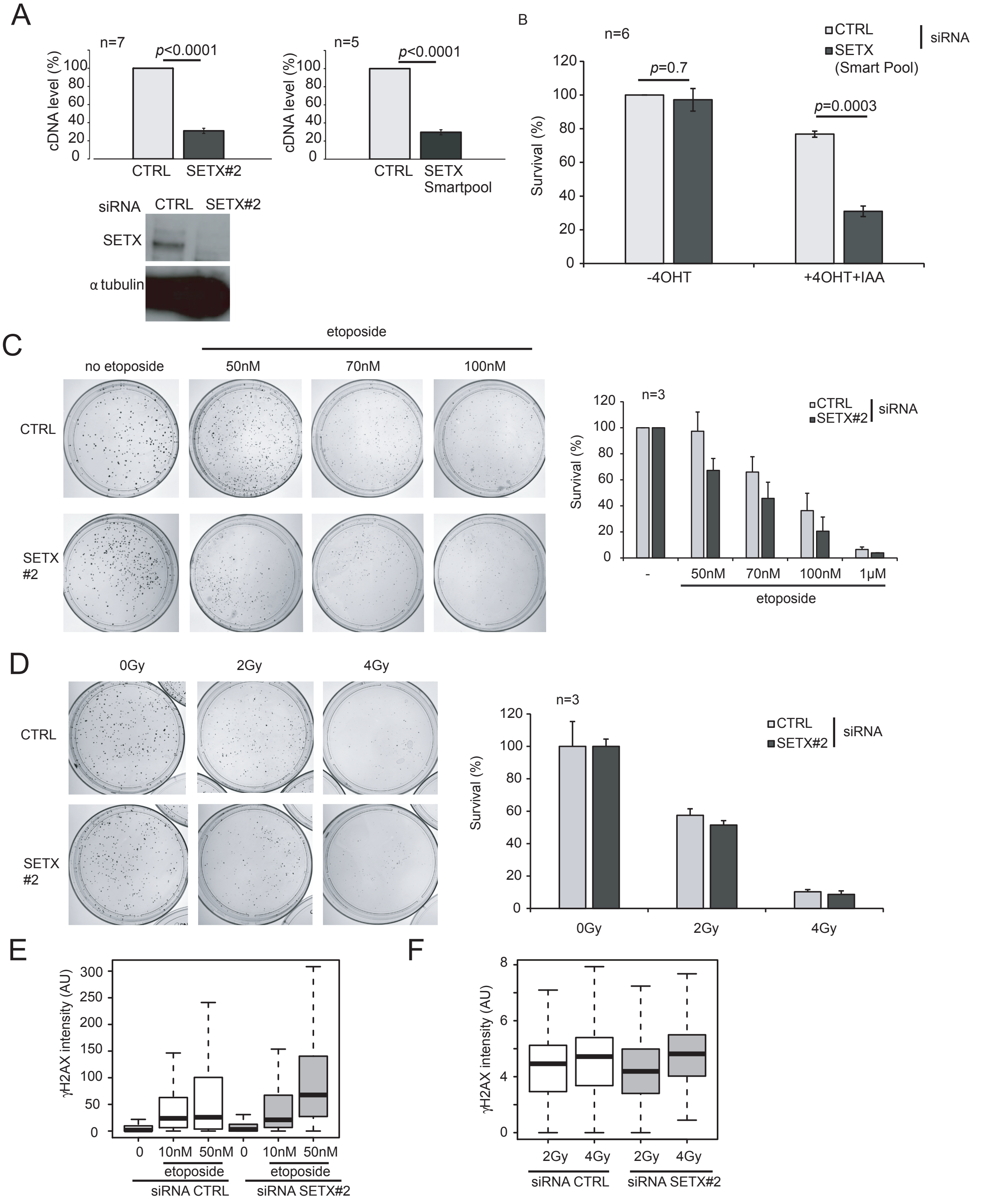

Supplementary Figure 3, related to Figure 3: Senataxin depletion triggers sensitivity to DSB induced by AsiSI and to etoposide but not to irradiation

A. cDNA levels, analysed by RT-qPCR in cells transfected with a control siRNA, a single siRNA directed against SETX (SETX#2 top panel) or a pool of siRNA directed against SETX (Smartpool, middle panel). mean and s.e.m of respectively 7 and 5 biological replicates are shown.Bottom panel shows a western blot in DIvA cells transfected with the control or SETX#2 siRNA. P-values are indicated (paired t-test). **B.** Clonogenic assays in AID-DIvA cells transfected with control and SETX smartpool siRNA, before (-4OHT) and after 4OHT treatment followed by auxin treatment (+4OHT+IAA) as indicated. Mean and s.e.m of 6 biological replicates are shown. P-values are indicated (paired t-test). **C.** Clonogenic assays in U2OS cells transfected with control and SETX #2 siRNA, following etoposide treatment as indicated. Mean and s.e.m of 3 biological replicates are shown. **D.** Clonogenic assays in U2OS cells transfected with control and SETX #2 siRNA, following exposure to γ-irradiation as indicated. Mean and s.e.m of 3 biological replicates are shown. **E**. Quantification of γH2AX signal detected in U2OS following treatment with increasing dose of etoposide, after transfection with control or SETX#2 siRNA as indicated. A representative experiment is shown (>100 nuclei). Center line: median; Box limits: 1st and 3rd quartiles; Whiskers: Maximum and minimum without outliers.**F**. Same as in E except that quantification was performed following exposure to 2 or 4Gy.

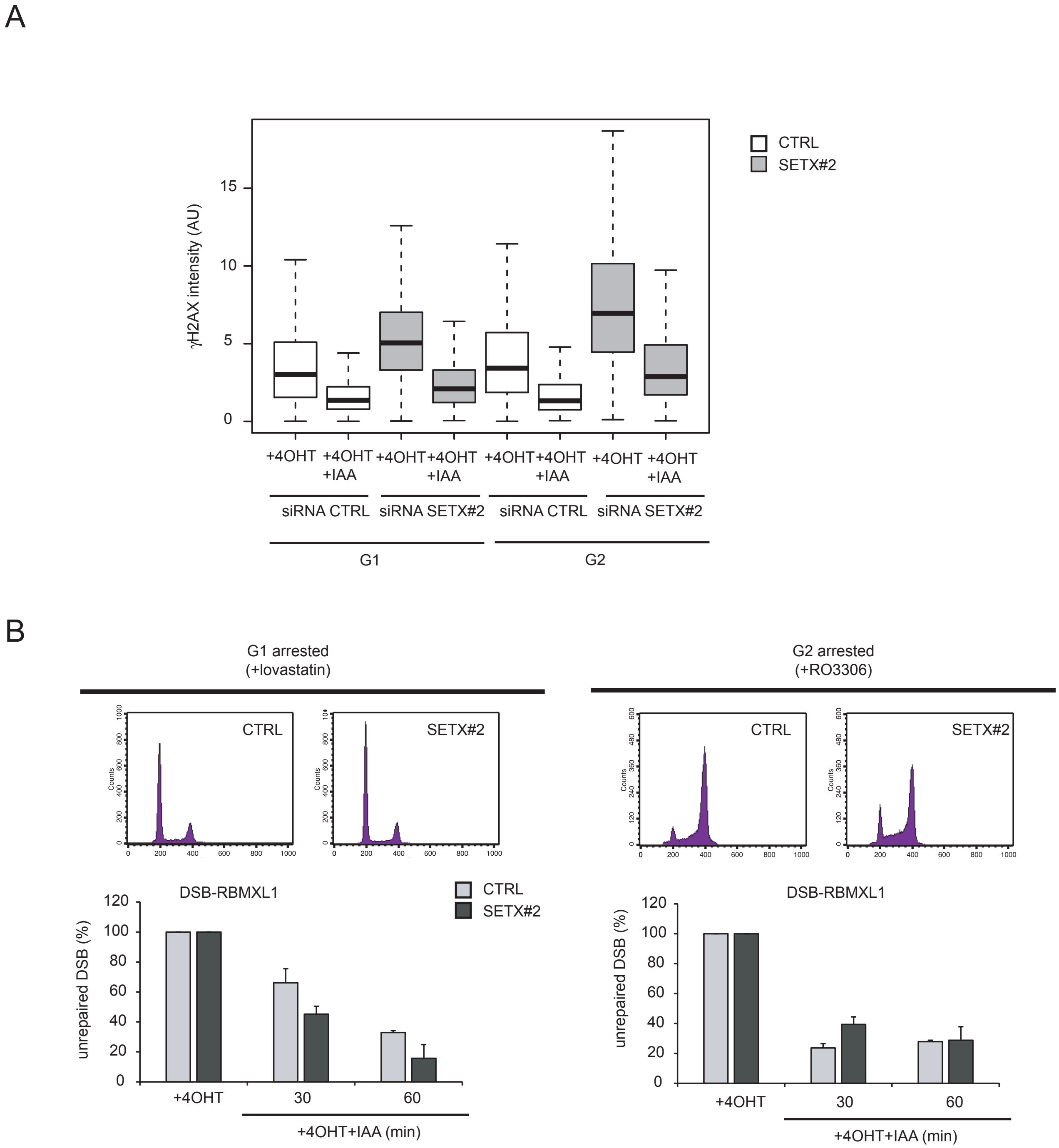

Supplementary Figure 4, related to Figure 4: Senataxin depletion does not delay repair kinetics in G1 and G2 cells
A. Quantification of γH2AX signal detected in AID DIvA cells following treatment with 4OHT (4h) and 4OHT followed by 2h of auxin (4OHT+IAA). Cells in G1 or G2 were sorted based on Hoechst staining, using an operetta device coupled to the Colombus software. A representative experiment is shown (>1000 nuclei). Center line: median; Box limits: 1st and 3rd quartiles; Whiskers: Maximum and minimum without outliers.**B**. Cleavage assay performed in AID-DIvA cells, arrested in G1 (left panel) or in G2 (right panel), left untreated or treated with 4OHT (4h) followed by auxin (IAA) addition (30min and 60 min), after transfection of control or SETX#2 siRNAs. Precipitated DNA was analyzed close to the DSB-RBMXL1, found to recruit Senataxin after 4OHT. The percentage of sites that remains broken after the indicated time of auxin treatment are presented. Mean and s.e.m of technical replicates from representative experiment are shown. FACS profiles are also shown (top panels) for both conditions.

A

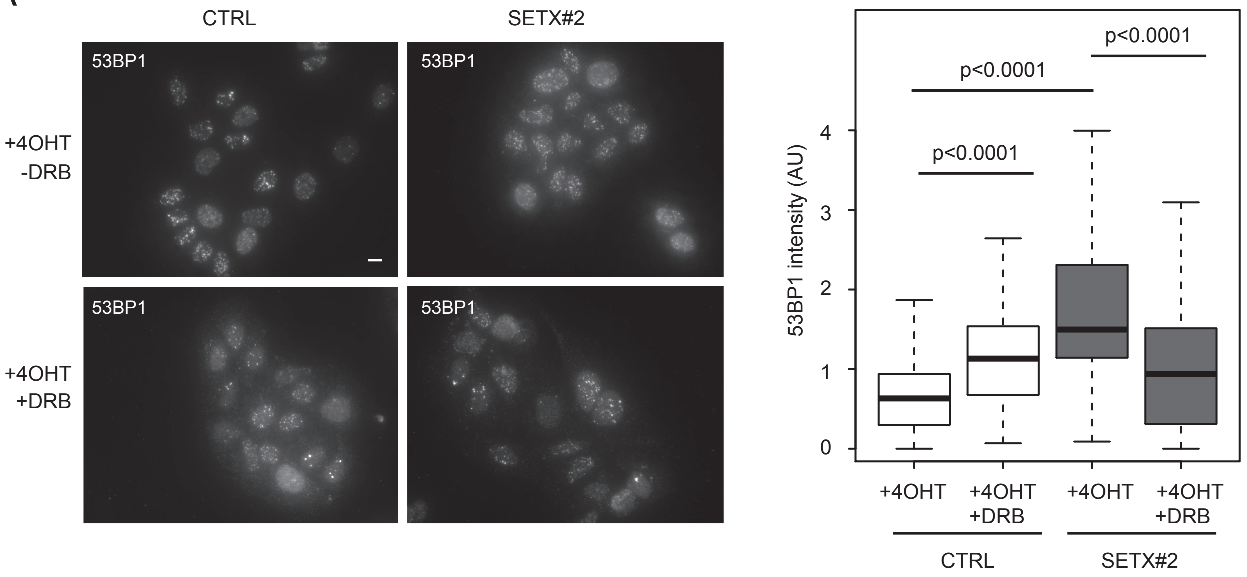

B

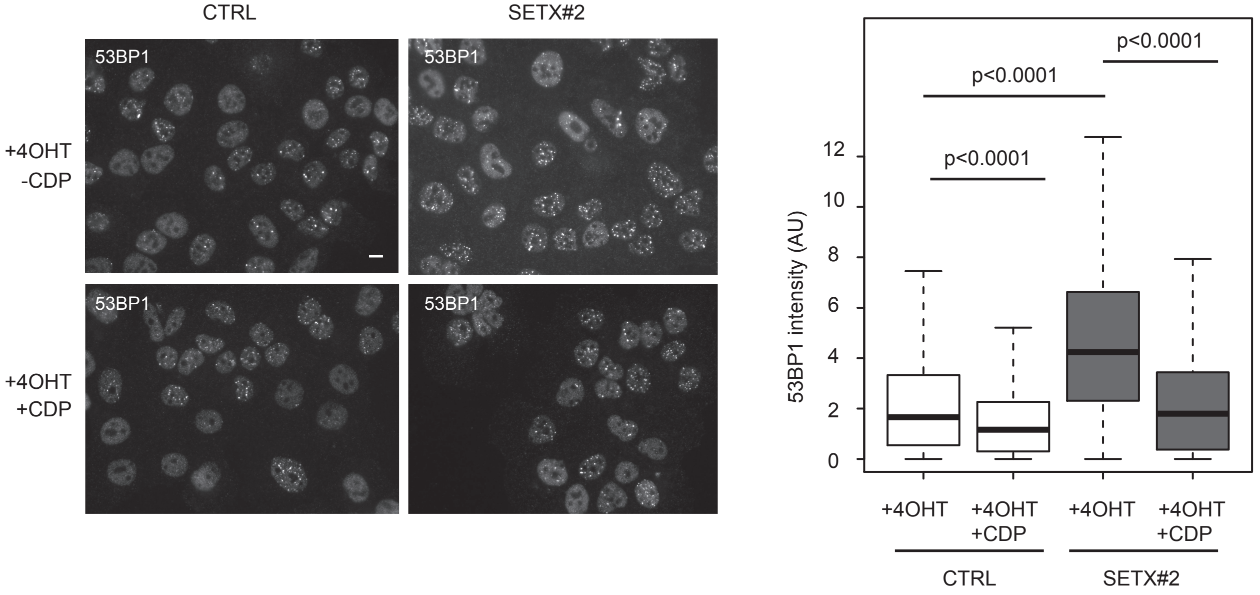

Supplementary Figure 5, related to Figure 5: Senataxin depletion increases 53BP1 foci formation in a manner partially reversed by prior exposition to transcription inhibitors.

A. 53BP1 staining performed in 4OHT- treated DIvA cells (4h), after transfection with control or SETX siRNA, in presence of DRB, a transcription inhibitor, as indicated. Right panel shows the quantification of the 53BP1 nuclear signal within foci (>100 nuclei) from a representative experiment. Center line: median; Box limits: 1st and 3rd quartiles; Whiskers: Maximum and minimum without outliers. P-values are indicated (unpaired t-test). **B**. Same as in A, except that a cordycepin pre-treatment was performed. Scale bar: 10μM

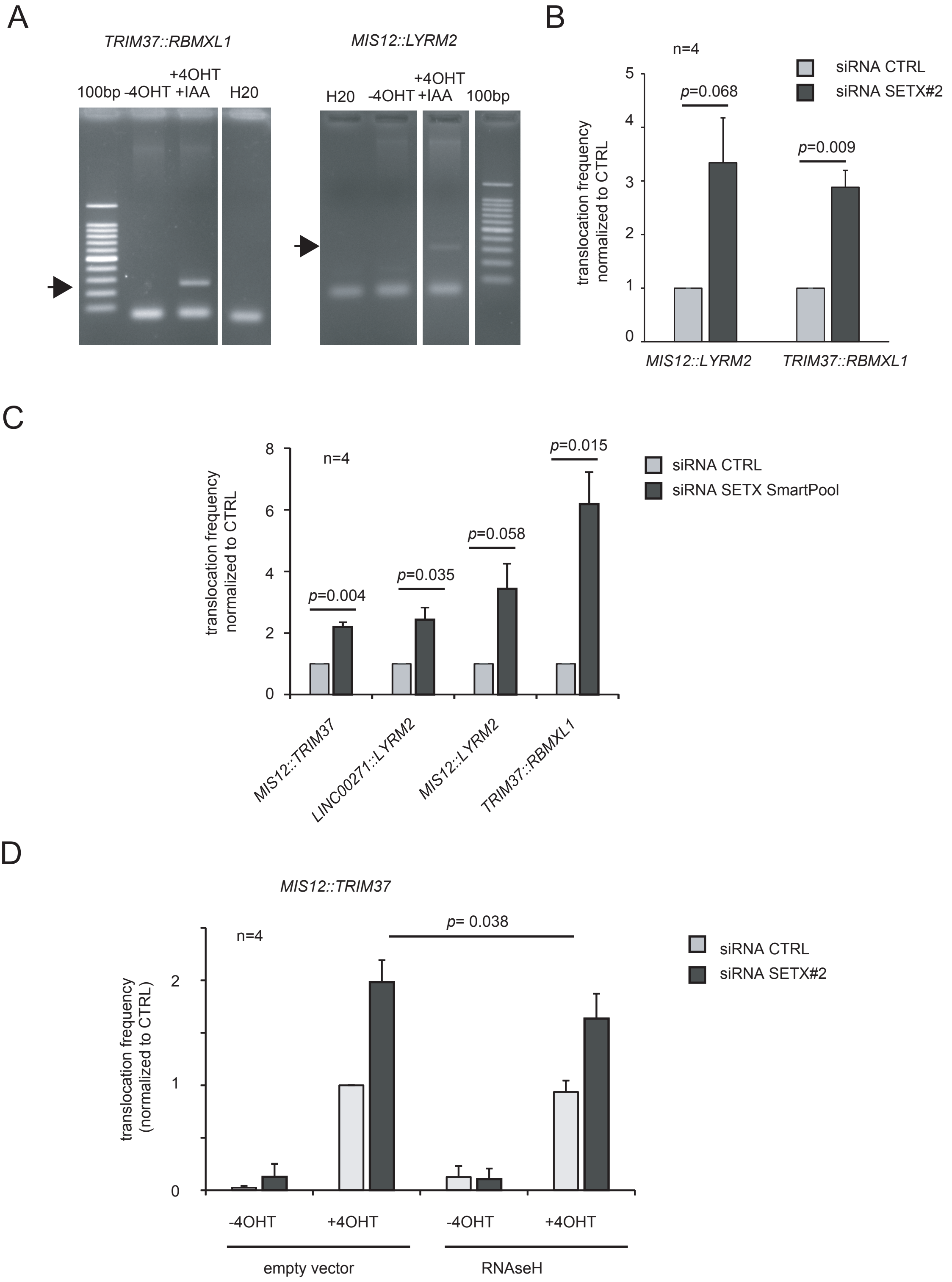

Supplementary Figure 6, related to Figure 6: Senataxin depletion increases translocation frequency in a manner partially reversed following overexpression of RNAseH1

A. Rejoining of distant DSBs located on different chromosomes were detected by PCR, following DSB induction and repair (+4OHT+IAA 2h). DNA sequencing confirmed the nature of the amplified products. **B**. *MIS12::LYRM2* and *TRIM37::RBMXL1* rejoining frequencies were analyzed after 4OHT+ IAA treatment, by quantitative PCR in AID-DIvA cells transfected with control or SETX directed siRNA (SETX#2). Data are normalized to the translocation level observed in control cells. Mean and s.e.m of 4 biological replicates are shown. *P* values are indicated (one sample t-test). **C.** All four translocations events were measured as above following depletion of senataxin using the SmartPool siRNA. Mean and s.e.m of 4 biological replicates are shown. *P* values are indicated (one sample t-test). **D**. *MIS12::TRIM37* rejoining frequency was analyzed in control or SETX-depleted AID-DIvA cells transfected with an empty vector or with a plasmid expressing RNAseH1, as indicated. Mean and s.e.m of 4 biological replicates are shown. *P* values is indicated (paired t-test).
